# Development of a Non-Target Screening and Quantitative Analysis Strategy Based on UPLC-Q-TOF/MS and UPLC-QQQ/MS to Improve the Quality Control of Wuling Capsule

**DOI:** 10.3390/molecules29112598

**Published:** 2024-05-31

**Authors:** Xiao-Feng Huang, Ying Xue, Jian Liang, Li Yong

**Affiliations:** 1 Chengdu Institute of Biology, Chinese Academy of Sciences, Chengdu 610041, China; huangxiaofeng493@163.com (X.-F.H.); liangjian@cib.ac.cn (J.L.); 2 Sichuan Center for Disease Control and Prevention, Chengdu 610041, China; xuecher0221@sina.com

**Keywords:** quality control, non-target screening, quantitative analysis, flavonoids, UPLC-Q-TOF/MS, UPLC-QQQ/MS

## Abstract

Herbal medicine has been widely valued because of its remarkable efficacy and minimal side effects. The quantitative analysis of herbal medicines is essential to ensure their safety and efficacy. The simultaneous detection of multiple quality markers (Q-markers) has emerged as an important approach and trend in herbal medicine quality control. In recent years, non-targeted screening has become an effective strategy for the discovery and identification of unknown compounds. This study developed a non-targeted screening and quantitative analysis strategy to discover, identify and quantify the multiple components that truly represent the efficacy of Wuling capsule. Within this strategy, 18 types of flavonoids were tentatively discovered and identified from Wuling capsule by analyzing mass cleavage pathways, the precise molecular weights of compounds, and comparing the data with a database. Ten types of flavonoids were determined after the comparison of the standards. Additionally, following the evaluation of the regression equation, linear range, limit of detection (LOD), limit of quantitation (LOQ), precision, repeatability, and recovery of the proposed quantitative method, six flavonoids were quantified. This method successfully screened, identified, and quantified the potential active components in Wuling capsule, providing insights for improving the quality control standards in other herbal medicines.

## 1. Introduction

Herbal medicine has been utilized for the clinical prevention and treatment of diseases for thousands of years. Its remarkable efficacy and minimal side effects have garnered increasing attention worldwide. Quantitative analysis is crucial to ensuring the efficacy and safety of herbal medicine [1]. Technological advancements have led to the use of various advanced instruments for the quality control of herbal medicine, including chromatographs [2], surface-enhanced Raman spectroscopy [3], capillary electrophoresis [4], near-infrared spectroscopy [5], mass spectrometry imaging [6], hyperspectral imaging [7], nuclear magnetic resonance (NMR) [8], microfluidics [9], etc. Among these, liquid chromatography with ultraviolet/mass spectrometry (LC-UV/MS) is the most widely used and highly accepted method. More importantly, the concept of ‘quality markers’ (Q-markers) has evolved from a single marker to comprehensive multi-markers [10]. These multi-markers often consist of structural analogues with the same skeleton, determined by the biogenic characteristics of plants. A prime example is the Chinese Pharmacopoeia (2020 edition), which designates five anthraquinones as the Q-markers for *Rhei Radix et Rhizoma* and four protoberberines as the Q-markers for *Coptidis Rhizoma*, respectively. The simultaneous detection of these multi-markers represents an important approach and trend in the quality control of herbal medicine.

Due to the synergistic effects of multiple components in herbal medicine, it is necessary to consider all the components as quality control markers [11]. However, a crucial prerequisite for achieving this goal is to determine the chemical components of herbal medicine through phytochemical separation, which is a traditional but time-consuming and laborious approach. Moreover, this method requires a large amount of harmful organic solvents. Therefore, it is urgent to develop a simple method that can efficiently discover structural analogues in herbal medicine for the quantification of multi-markers in quality control.

Non-target screening has emerged as an effective strategy in recent years for the discovery and identification of analogues in plants. This approach utilizes omics methods or established databases to screen and identify unknown compounds through extensive and non-selective data collection [12,13]. The combination of liquid chromatography and high-resolution mass spectrometry (HRMS) has greatly facilitated non-targeted screening, as it allows for the collection of valuable compound information such as the retention time, accurate molecular weight, and elemental composition of fragment ions. This information proves helpful in the structural identification of unknown compounds even in the absence of authentic standards [14,15,16]. When compared to authentic standards, compounds can be unambiguously identified for quantitative analysis [17].

*Xylariasp* is the sclerotium of the fungus *Xylaria nigripes* and exhibits various biological activities such as the regulation of signal pathways, the enhancement of neurotransmitter activity, and neuroprotection [18,19,20]. It has been formulated into Wuling capsule for the treatment of insomnia disorder, as well as the improvement of depression and cognitive function in clinical practice [21,22,23]. The Chinese Pharmacopoeia (2020 edition) designates mannitol and adenosine as the quantitative markers for Wuling capsule. Recently, our team identified an additional nine types of nucleosides in Wuling capsule [24]. However, as primary metabolites, nucleosides cannot directly represent the efficacy of the medicine. Therefore, it is necessary to discover and select secondary metabolites with pharmacological activity as multi-markers for quality control. Previous reports have identified genistein, a type of flavonoid, as a quantifiable compound in Wuling capsule [25,26]. As an important secondary metabolite of plants, flavonoids have been proven to protect nerve cells from damage and delay neurodegenerative diseases in previous studies, which is closely related to the therapeutic application of Wuling capsule [27,28,29].

Thus, this study aims to establish a rapid and sensitive non-targeted screening and quantitative analysis strategy based on ultra-high-performance liquid chromatography with quadrupole time-of-flight mass spectrometry (UPLC-Q-TOF/MS) and ultra-high-performance liquid chromatography with triple quadrupole mass spectrometry (UPLC-QQQ/MS) technology to screen, identify and quantitatively analyze multiple potential active flavonoids in Wuling capsule, and provide insights for improving the quality control standards of other herbal medicines.

## 2. Results and Discussion

### 2.1. Non-Target Screening and Identification of Flavonoids by UPLC-Q-TOF/MS

The systematic identification workflow was carried out in the following three steps. The compounds were initially screened by comparing the mass spectrum information collected in data-dependent detection with the mass spectrum data in three libraries (i.e., PubChem, NIST/EPA/NIH Mass Spectral Library and MassBank). Then, the data were manually analyzed to identify the potential flavonoids based on the fragmentation characteristics of the mass spectrum. Finally, the exact structure of the flavonoids was identified by comparing the retention time with the authentic standards.

#### 2.1.1. Tentative Identification of Flavonoids

The methanol extract of Wuling powder was analyzed by full scanning in the UPLC-Q-TOF/MS positive and negative ion mode. Peak View 2.0 software was used for the data analysis and library search to tentatively screen for suspected compounds. After extensive data collection in the corresponding detection mode, the chromatographic peaks with a signal intensity ≥50 counts or a signal-to-noise ratio >3 in the full range were automatically extracted using the non-targeted peak-finding mode. The following settings were used: *m*/*z* tolerance width ±0.02 Da, retention time tolerance ±0.4 min, threshold value 10 cps, intensity threshold value 0.05. The exact molecular weight, retention time, isotope ratio, molecular formula, MS spectrum, MS/MS spectrum, and elemental composition of the fragment ions of the compounds were obtained, which were used as the basis for subsequent identification. Among them, the screening results with an accurate molecular weight calculation error within ±5 ppm, a retention time error less than 2%, an isotope ratio error less than 10%, a library score and formula finder score both above 70 were acceptable.

**Figure 1:**
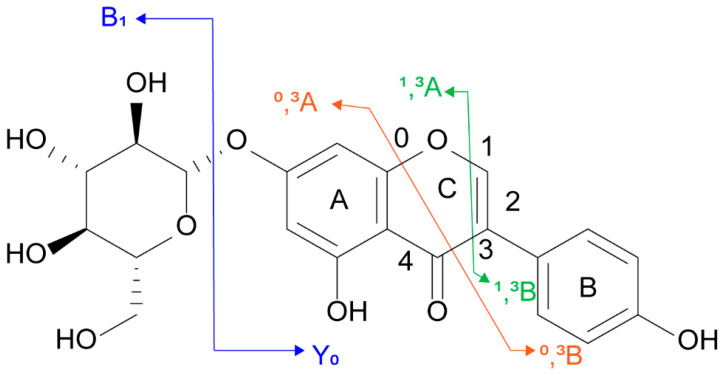
Fragmentation nomenclature used for genistin **(2)**.

Subsequently, the typical mass spectrometric cleavage pathway of flavonoids was used as the main basis for the tentative discovery and identification. For example, flavonoid *O*-glycosides can generate a high abundance of flavonoid aglycone moieties mainly through the cleavage of glycosidic *O*-linkages [30]. The fragmentation patterns of the aglycone moiety mainly include RDA reaction, cross-ring cleavage pathway, and loss of CO (−28), H_2_O (−18), CH_3_ (−15), OH (−17), and other fragments to generate fragment ions [17,31]. The feasibility of the identification method was demonstrated by analyzing the cleavage pathway of authentic standards. The fragment ions of flavonoids were named according to the rules proposed by Ma et al., as shown in Figure 1 [32].

**Figure 2:**
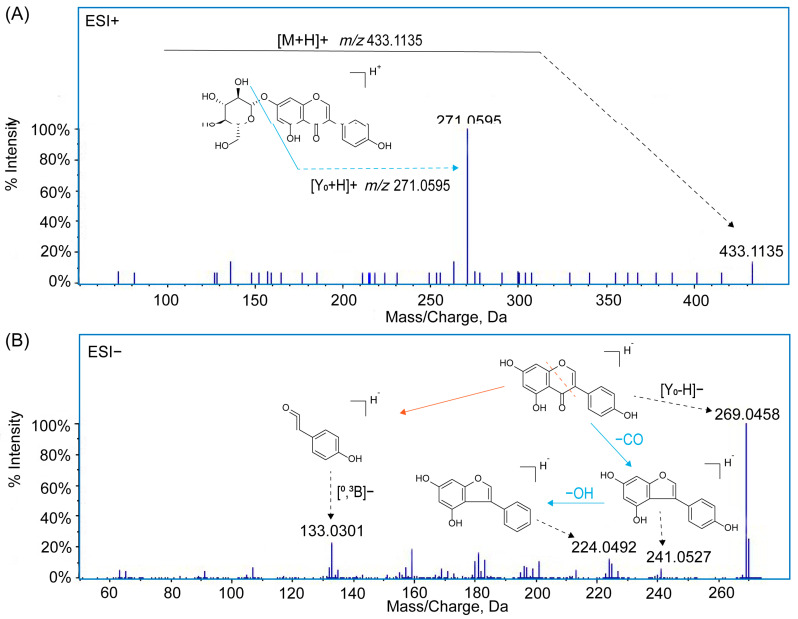
MS/MS spectra and the main cleavage pathways in corresponding modes of compounds (**A**): genistin **(2)** in the ESI^+^ mode; (**B**): genistin **(14)** in the ESI^−^ mode.

For example, in the positive ion mode, a deprotonated ion with an exact mass number of *m*/*z* 433.1135 [M+H]^+^ was detected. The fragment ion *m*/*z* 271.0595 [Y_0_+H]^+^ was produced by the loss of one mol of C_6_H_10_O_5_ (162 Da) from the deprotonated ion. Thus, the compound was inferred to be genistin **(2)**, which lost a molecule of glucose after the glycosidic *O*-linkages was broken, and the aglycone moiety was formed. In the negative ion mode, the ion *m*/*z* 269.0458 [Y_0_-H]^−^ was detected. The fragment ion *m*/*z* 241.0506 [Y_0_-H-CO]^−^ was generated by the loss of a molecule of CO from [Y_0_-H]^−^, and the further loss of OH generated the fragment ion *m*/*z* 224.0492 [Y_0_-H-CO-OH]^−^. The fragment ion *m*/*z* 133.0301 [^0^,^3^B_0_]^−^ was generated by the cross-ring cleavage of [Y_0_-H]^−^, so the compound was identified as genistein **(14)**. The fragmentation pathways have also been verified by authentic standards. The corresponding MS/MS spectra and cleavage pathways are shown in Figure 2.

Acetyldaidzin **(3)**, was identified by the mass spectrometric cleavage pathway combined with biogenesis prediction and comparison with data from the literature [33,34]. According to the above identification methods, 18 types of flavonoids were tentatively discovered and identified, and their basic information is presented in Table 1.

#### 2.1.2. Identification of Known Flavonoid

**Figure 3:**
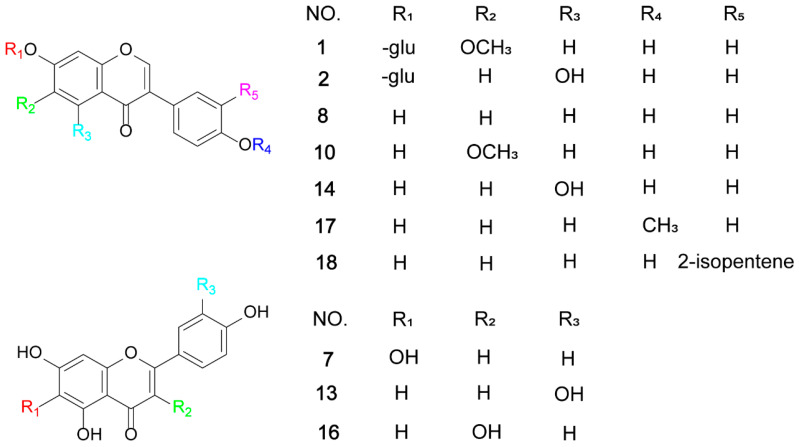
The chemical structure of known flavonoids.

Due to the complex composition of flavonoids in the extract and the presence of various isomers with different substitution patterns, it is difficult to accurately identify them only by mass spectrometric characters. Therefore, the structure of flavonoids in Wuling powder was further confirmed by comparing with the authentic standards. By comparing the retention time and tandem mass spectrometry with the standards, ten flavonoids, including seven aglycones and three glycosides, were clearly identified: glycitin **(1)**, genistin **(2)**, scutellarein **(7)**, daidzein **(8)**, glycitein **(10)**, luteolin **(13)**, genistein **(14)**, kaempferol **(16)**, formononetin **(17)**, and neobavaisoflavone **(18)**. The chemical structure of all the known flavonoids is shown in Figure 3.

### 2.2. Quantification of Six Flavonoids by UPLC-QQQ/MS

#### 2.2.1. Optimization of the UPLC-QqQ/MS Conditions

In this study, UPLC-QQQ/MS was used to quantitatively analyze multiple flavonoids in the multiple reaction monitoring (MRM) mode based on the results of the non-targeted screening. MRM is a powerful target MS spectrometry technique that can improve the sensitivity and accuracy of the quantification of components in complex samples by excluding the interference of impurity ions through characteristic ion selection [35]. In addition, the mobile phase gradient and MS parameters of the analytical method were adjusted to obtain better peak shapes and response values. After preliminary testing, six flavonoids high in content, glycitin **(1)**, genistin **(2)**, daidzein **(8)**, glycitein **(10)**, genistein **(14)**, and formononetin **(17)**, could be accurately quantified above the limit of detection of mass spectrometry. The mass spectrometry cleavage parameters of the six flavonoids are shown in Table 2.

#### 2.2.2. Method Validation

The quantitative method was validated using standard solutions under the described UPLC-QQQ/MS conditions. Various analysis parameters, such as the linearity, limit of detection (LOD), limit of quantification (LOQ), precision, repeatability, and recovery, were assessed. The calibration curves of the six analytes were generated using the mixed working solutions II–XI. The linear regression equations were obtained by plotting the concentration of each authentic standard as the abscissa (*x*) and the corresponding experimental peak area as the ordinate (*y*). Correlation coefficient values (R^2^) of above 0.9995 were obtained for all the analytes, indicating a good correlation between the experimental data and the theoretical ratios. The sensitivity was evaluated by measuring the LOD at a signal-to-noise ratio (S/N) of 3:1, and an LOQ at S/N of 10:1. To validate the precision of the proposed method, the relative standard deviations (RSDs) of the peak areas of three concentration quality control (QC) solutions (low, middle, high) were calculated for intra-day and inter-day variations. The intra-day precision was measured by analyzing the QC solutions in triplicate within one day, and the inter-day precision was tested by analyzing the same solutions on three different days. The repeatability was evaluated by analyzing six independent portions of sample 230110. The recovery was assessed by adding standards at a ratio of approximately 1:1 to 230110 samples halved in mass and performed in parallel operation six times. The results, summarized in Table 3, show that the analytical method meets the requirements of quantitative analysis and is suitable for the quantification of the six flavonoids in Wuling powder.

#### 2.2.3. Quality Evaluation of Wuling Samples

The proposed UPLC-QQQ/MS method was utilized to quantify six flavonoids in two forms of commercially available Wuling capsule, and the results are shown in Table 4. Considering the large difference in the content of flavonoids in samples, the extract solution was analyzed without dilution and after dilution of five times in the sample pretreatment stage to ensure the quantification of each compound within their respective linear range. The findings reveal that the flavonoids content of in different batches of Wuling powder, from high to low, is as genistein **(14)**, daidzein **(8)**, glycitein **(10)**, glycitin **(1)**, genistin **(2)** and formononetin **(17)**. The content of the other five flavonoids (daidzein **(8)**, glycitein **(10)**, glycitin **(1)**, genistin **(2)** and formononetin **(17)**), except genistein **(14)** were quantified in Wuling powder for the first time. Although the content of genistein **(14)** is the highest one, the content of daidzein **(8)** and glycitein **(10)** is also relatively high. Therefore, we believe that daidzein **(8)**, glycitein **(10)** and genistein **(14)** should be designates as Q-marker to better supervise the quality of Wuling capsule.

## 3. Material and Methods

### 3.1. Chemicals and Reagents

Ten authentic standards were purchased from their respective companies: glycitin (CAS: 40246-10-4)) (Beijing Shiao Biological Technology Co., Ltd., Beijing, China, ≥98%, genistin (CAS: 529-59-9), glycitein (CAS: 40957-83-3), neobavaisoflavone (CAS: 41060-15-5) (Wuhan Zhongbiao Biological Technology Co., Ltd., Wuhan, China, 95%~99%,), daidzein (CAS: 486-66-8) (Tianjin Heowns Biochemical Technology Co., Ltd., Tianjin, China, 98%), luteolin (CAS: 491-70-3), genistein (CAS: 445-72-0) (Shanghai Dibo Biological Technology Co., Ltd., Shanghai, China, ≥98%), kaempferol (CAS: 520-18-3), scutellarein (CAS: 529-53-3), formononetin (CAS: 485-72-3) (Sichuan Weikeqi Biological Technology Co., Ltd., Chengdu, China, ≥98%). Two batches of Wuling capsule (batch number 210117, 230110) were obtained from Zhejiang Jolly Pharmaceutical Co., Ltd., Huzhou, China. The ultra-pure water was prepared using the Milli-Q water purification system (Millipore, Bedford, MA, USA). The methanol (CAS: 67-56-1) with LC-MS grade were purchased from Sigma-Aldrich (Sigma-Aldrich, St. Louis, MO, USA).

### 3.2. Standard Solution Preparation

Stock solutions of 1 mg/mL of glycitin **(1)**, genistin **(2)**, daidzein **(8)**, glycitein **(10)**, genistein **(14)**, and formononetin **(17)** were obtained by dissolving approximately 10 mg of each authentic standard in a 10 mL brown volumetric flask with methanol. Subsequently, 250 μL of each of the six stock solutions was transferred to a 25 mL brown volumetric flask and diluted with methanol to the scale line to obtain the mixed working solution I with a concentration of about 10 µg·mL^−1^. Mixed working solution I was diluted by gradient to obtain mixed working solutions II–Ⅺ with concentrations of about 1~1000 ng·mL^−1^. Quality control (QC) solutions with low, medium, and high concentrations were also prepared by diluting working solution I. All solutions were stored at 4 °C before use.

### 3.3. Sample Preparation

A total of 150 mg Wuling powder was placed into a 10 mL brown volumetric flask and accurately weighted. Methanol was added to reach the scale line, and the mixture was sonicated for 1 h (100 W, 40 kHz). The volume was fixed again with methanol to reach the scale line after the sample was cooled to room temperature. Finally, the solutions were filtered through a 0.22 µm syringe filter. The filtrate and a five-fold diluted filtrate were injected into UPLC spectrometer for analysis.

### 3.4. Non-Target Screening of Flavonoids by UPLC-Q-TOF/MS

The Shimadzu UPLC system (Shimadzu, Japan) consists of pumps (LC-30AD), an online degasser (DGU-20A5R), an auto-sampler (SIL-30AC), and a column oven (CTO-30aHE). Chromatographic separation was performed on an ACQUITY UPLC HSS T3 analytical column (2.1 mm × 100 mm, 1.8 µm) at 40 °C. The mobile phase consisted of an aqueous phase (0.1% formic acid) and an organic phase (methanol). The constant flow rate was 0.4 mL·min^−1^. The linear gradient elution was 5%~5%~95%~95%~5%~5% organic phase at 0~1~19~21~24~30 min. A volume of 1 µL of sample solution was injected into the UPLC system by the auto-sampler.

The TOF mass spectrometry instrument was a 4600 Q-TOF mass spectrometer (AB Sciex, Concord, CA, USA) equipped with an ESI source. The mass scanning was performed in positive and negative mode with the following parameters: ion source gas1 (N2) at 50 psi, ion source gas2 (N2) at 50 psi, curtain gas at 35 psi, temperature at 500 °C, ion-spray voltage floating at 5000 V and −4500 V, and declustering potential at 80 V, collision energy at 10 eV of MS1, collision energy at 35 eV of MS2. The full scan range was set to *m*/*z* 100~2000 Da. The system was operated under Analyst 1.6 (AB Sciex, Concord, CA, USA). An APCI positive and negative calibration solution was used to calibrate the instrument’s mass accuracy in real time.

### 3.5. Determination of Flavonoids by UPLC-QQQ/MS

Chromatographic separation was performed on a Waters ACQUITY H UPLC system (Waters, Milford, MA, USA) under the same analytical column and mobile phase as those used in the UPLC-Q-TOF/MS. The linear gradient elution was adjusted as follows: 30%~30%~60%~80%~90%~90%~30%~30% organic phase at 0~0.5~1.5~6.5~7.0~9.0~9.5~10.0 min for the quantification of flavonoid aglycones, 5%~5%~12%~40%~80%~80%~5%~5% organic phase at 0~0.5~2.0~5.0~8.0~11.0~11.5~12.0 min for the quantification of flavonoid glycosides. The constant flow rate was 0.4 mL·min^−1^. A volume of 2 µL of sample solution was injected into the UPLC system by the auto-sampler.

The QQQ/MS instruments was a 3200 Q-TRAP mass spectrometer (AB Sciex, USA) equipped with an ESI source. The quantitative analysis was performed in positive (flavonoid glycosides) and negative (flavonoid aglycones) ion mode under multi-reaction monitoring (MRM) mode. The MS parameters were as follows: ion source gas1 (N2) at 55 psi, ion source gsa2 (N2) at 55 psi, curtain gas at 25 psi, temperature at 550 °C, ion-spray voltage at 5500 V and −4500 V. Data collection and processing were conducted with Analyst software 1.6 (AB Sciex, USA).

## 4. Conclusions

In this study, a non-targeted screening method based on high-resolution UPLC-Q-TOF/MS was successfully established for efficient and comprehensive screening and identification of multiple potential active components in Wuling capsule. Through library matching and MS cleavage pathway analysis, 18 flavonoid components were tentatively identified. After comparing with authentic standards, 10 specific flavonoids were successfully identified. It can be seen that some of the compounds in Table 1 are identified as “unknown”. Even though they are involved in the cleavage pathway of flavonoids, they do not match with the results of the library search. In addition, some of them are isomers with the same fragment ions, which cannot be accurately identified because authentic standards are not available. In the future, the identification of these unknown compounds will help further explore the pharmacologically active ingredients of Wuling capsule. On the basis of non-targeted screening and identification, a highly sensitive quantitative analysis method based on UPLC-QQQ/MS was developed for the rapid and accurate quantification of six flavonoids in two forms of commercially available Wuling capsule. It is worth noting that in addition to genistein, the other five flavonoids, which truly represent the pharmacological activity of Wuling capsule, were quantified for the first time. The results highlight the effectiveness of combining UPLC-Q-TOF/MS non-targeted screening with UPLC-QQQ/MS quantitative analysis as a strategy to discover and identify multiple Q-markers, thereby enhancing the comprehensive quality control of herbal medicine.

## Figures and Tables

**Table 1:** The information of flavonoids in the extract of Wuling powder by UPLC-Q-TOF/MS.

NO.	Retention Time (min)	ESI Mode	Molecular Formula	Theoretical (*m*/*z*)	Experimental (*m*/*z*)	Error (ppm)	Fragment Ions (*m*/*z*)	Tentative Identification
1	10.56	[M+H]^+^	C_22_H_22_O_10_	447.1286	447.1297	2.6	285.0764, 270.0562	Glycitin
2	11.29	[M+H]^+^	C_21_H_20_O_10_	433.1129	433.1135	1.3	271.0595, 215.0702, 159.0428	Genistin
3	11.57	[M+H]^+^	C_23_H_22_O_10_	459.1286	459.1289	0.7	255.0652, 227.0679, 199.0759	Acetyldaidzin
4	11.81	[M−H]^−^	C_16_H_12_O_6_	299.0561	299.0564	0.9	284.0343, 256.0352, 239.0336, 212.0489, 200.0487, 148.0142	Unknown
5	12.27	[M−H]^−^	C_15_H_10_O_6_	285.0405	285.0413	3	241.0523, 213.0551, 185.0605, 171.0589, 156.0625	Unknown
6	12.59	[M−H]^−^	C_16_H_12_O_6_	299.0561	299.0565	1.1	284.0339, 256.0383, 231.0223, 210.9797, 192.9927,183.0437, 166.9822, 154.9918	Unknown
7	13.23	[M−H]^−^	C_15_H_10_O_6_	285.0405	285.0403	−0.5	257.0460, 241.0492, 229.0489, 212.0505	Scutellarein
8	13.4	[M−H]^−^	C_15_H_10_O_4_	253.0506	253.0507	0.3	224.0484, 208.0539, 196.0523, 180.0589, 133.0297, 91.0197	Daidzein
9	13.42	[M−H]^−^	C_19_H_16_O_7_	355.0823	355.0822	−0.4	314.9869, 295.0048, 253.0489, 231.0052, 211.0426, 135.0046	Unknown
10	13.66	[M−H]^−^	C_16_H_12_O_5_	283.0612	283.0616	1.6	268.0375, 240.0425, 211.0388, 196.0531, 184.0518	Glycitein
11	13.82	[M+H]^+^	C_17_H_14_O_6_	315.0863	315.0868	1.5	297.0441, 255.0649	Unknown
12	13.99	[M+H]^+^	C_19_H_16_O_7_	357.0969	357.0966	−0.7	311.0981, 255.0647, 237.0502, 199.0781, 181.0615, 137.0278	Unknown
13	14.11	[M−H]^−^	C_15_H_10_O_6_	285.0405	285.0407	0.9	241.0488, 213.0559, 187.0397, 157.0663, 145.0631, 123.0094, 95.0146	Luteolin
14	14.2	[M−H]^+^	C_15_H_10_O_5_	269.0456	269.0458	0.9	241.0527, 224.0492, 133.0301, 107.0149	Genistein
15	14.21	[M−H]^−^	C_16_H_12_O_6_	299.0561	299.0563	0.5	284.0358, 255.0350, 183.0465, 137.0032	Unknown
16	14.63	[M−H]^−^	C_15_H_10_O_6_	285.0405	285.0410	1.8	257.0466, 229.0519, 185.0595, 149.0255	Kaempferol
17	15.29	[M−H]^−^	C_16_H_12_O_4_	267.0663	267.0665	1	252.0431, 223.0404, 208.0526, 132.0238	Formononetin
18	16.69	[M−H]^−^	C_20_H_18_O_4_	321.1132	321.1135	0.9	252.0434, 223.0396, 195.0456	Neobavaisoflavone

**Table 2:** UPLC-QQQ/MS parameters of six flavonoids.

Analyte	Retention Time (min)	Detection Mode	Parent Ion (*m*/*z*)	Product Ion (*m*/*z*)	DP (V)	CE (eV)
Glycitin **(1)**	6.24	[M+H]^+^	447	385 ^#^, 285 *	39	26, 28
Genistin **(2)**	6.63	[M+H]^+^	433	271 ^*#^	52	41
Daidzein **(8)**	3.12	[M−H]^−^	253	132 *, 91 ^#^	−66	−46, −53
Glycitein **(10)**	3.20	[M−H]^−^	283	268 *, 240 ^#^	−50	−24, −34
Genistein **(14)**	3.49	[M−H]^−^	269	159 ^#^, 133 *	−65	−34, −42
Formononetin **(17)**	4.20	[M−H]^−^	267	252 *, 223 ^#^	−60	−29, −45

Notes: * quantitative ion, ^#^ qualitative ion.

**Table 3:** The results of method validation.

Analyte	Regression Equation, *r*	Linear Range (ng∙mL^−1^)	LOD (ng∙mL^−1^)	Precision (RSD)						Repeatability (*n* = 6)		Recovery (*n* = 6)	
				Intra-Day (%) (*n* = 6)			Inter-Day (%) (*n* = 18)			Mean (μg∙g^−1^)	RSD (%)	Mean (%)	RSD (%)
				Low	Medium	High	Low	Medium	High				
Glycitin **(1)**	*y* = 66.98*x* + 328.49, 0.9998	9.6~960	4.80	3.80	2.47	1.84	4.23	3.45	2.71	8.77	3.40	99.02	4.73
Genistin **(2)**	*y* = 88.328*x* + 66.149, 0.9999	9.3~930	2.33	4.46	2.75	1.02	4.66	3.20	1.40	0.93	3.84	97.48	3.93
Daidzein **(8)**	*y* = 135.86*x* + 81.416, 0.9998	2.55~255	1.02	3.59	2.95	1.85	4.51	2.36	2.49	70.72	3.94	100.41	2.70
Glycitein **(10)**	*y* = 782*x* − 146, 0.9999	1.02~102	0.25	4.20	3.17	0.72	4.81	3.55	1.64	32.85	2.47	95.66	4.54
Genistein **(14)**	*y* = 207.22*x* − 11.976, 0.9999	4.85~970	2.43	1.87	3.37	1.55	3.60	4.71	2.99	154.88	1.17	104.51	3.53
Formononetin **(17)**	*y* = 620.1*x* + 323.56, 0.9998	0.95~47.5	0.48	3.79	1.14	0.87	4.23	3.11	2.43	0.17	4.89	95.90	4.54

**Table 4:** The contents of six isoflavones in two forms of Wuling capsule (μg·g^−1^).

Batch	Glycitin (1)	Genistin (2)	Daidzein (8)	Glycitein (10)	Genistein (14)	Formononetin (17)
210117	6.21	0.63	107.55	29.06	191.38	0.12
230110	8.75	0.95	69.08	31.71	157.57	0.18

## Data Availability

Data are contained within the article.
